# Supplementary material for: Forecasting ocean acidification impacts on kelp forest ecosystems
Source: PLoS One. 2021 Apr 22;16(4):e0236218. doi: 10.1371/journal.pone.0236218 (PMC8061940; doi:10.1371/journal.pone.0236218)
Supplement: Attachment — Submitted filename: Review PLoS Schlenger.docx [file pone.0236218.s001.docx]

Review for: “Forecasting ocean acidification impacts on kelp forest ecosystems” by Schlenger et al. Overall, the paper was well written and understandable.

Using ecological models to integrate results from different methods. Thus leading to an assessment of Ocean Acidification on kelp community structure. Better understanding of food web dynamics responding to external forcing parameters. Present a quantitative modeling approach to asses the cumulative effects of multiple OA impacts on ecosystem dynamics. Results reveal that impacts from OA is strongly influenced by specific physiological interactions of OC with specific functional groups and species.

Overall comments

Overall, the manuscript is well written and the research is interesting and validates previous similar studies.

I recommend to the authors to read and include the Minireview by Harley et al. 2012 “Effects of climate change on global seaweed communities” – it has some very relevant insight.

To bottom of page 27- the authors could add more detailed data from Kubler et al. 2015 PLoS ONE “Predicting effects of ocean acidification and warming on algae lacking carbon concentrating mechanisms.”

Specific comments

A map of Isla Navidad would be useful to add to the intro – under “ecopath Model”

Figure 1 – the text is very blurry – please make the font bigger and more legible. Figure 2 is also blurry – please make more legible.

Table 1 . “Sea palm” usually refers to *Postelsia palameformis*. Curious on why do you separate sea palm from brown algae and *Sargassum* categoryTechnically, *Sargassum*, *Macrocystis*, *Ecklonia* are all brown algae too. Throughout the text and in your figures, you predominantly refer to brown algae – so it is not clear what groups you include. Perhaps there is a better way to make the distinction?

Pg 18 – missing % after 15.

Figure 2 – why only “other mortality” where is overall mortality (natural + predation +fishing +other)? Is it included in Vulnerability?
