# Supplementary material for: Forecasting ocean acidification impacts on kelp forest ecosystems
Source: PLoS One. 2021 Apr 22;16(4):e0236218. doi: 10.1371/journal.pone.0236218 (PMC8061940; doi:10.1371/journal.pone.0236218)
Supplement: Attachment — Submitted filename: Schlenger et al. Response to Reviewers.docx [file pone.0236218.s002.docx]

**Reviewer #1: This study presents an interesting analysis of the impact of OA on a kelp forest system based on robust modeling techniques, and the authors wrote it well except some careless grammar errors or typos. While previous studies have used EwE to assess the ecological impacts of OA, few have focused specifically on nearshore coastal kelp ecosystems or quantified the role of different physiological forcing mechanisms. So this study is novel and well address this gap. However, there are some concerns relating to methods and interpretation of the results (shown below) which I suggest the authors pay attention to and revise the paper accordingly.**

**General comments:**

**1. It might serve the paper better if there are some analyses of the interactive effect of the three forcing types. For instance, the authors could construct another model which only add production and vulnerability forcing to the model and compare the result with the two individual ones mathematically.**

Although we agree with the reviewer that an exploration of the interactive effects between each forcing type would be a useful exercise to better understand the model behavior, we feel that the interpretation of those results would not be ecologically relevant. In a natural setting, there would not be an instance where only two of these mechanisms would be acting in conjunction with each other at the ecosystem-level. Furthermore, the novel utility of this study lies in taking these individual forcing mechanisms, previously looked at independently in other publications, and understanding their combined dynamics in an effort to step as close to ecological realism as possible. We feel that only looking at combinations of two mechanisms at a time would not provide useful insights for those trying to replicate this method in other models or those attempting to validate these findings in the field.

**2. I found the authors may need to pay attention to the tense of their writing in Results. Using past tense for describing results is a norm in academic literature.**

The verb tense has been changed to past tense as appropriate.

**3. The authors should be more careful in interpreting their results in their writing of Abstract and Results. As I found multiple times that there is overinterpretation (e.g. generally consistent which is in fact not so true). I suggest they would make the paper more interesting to highlight the different response of the ecosystem or populations to different OA forcing mechanisms.**

We have added a number of revisions clarifying the interpretation of results or adding context highlighting uncertainties in those interpretations. A more detailed breakdown of those changes can be found below.

We have also described a wide variety of changes in abundance across groups and species as well as the ecosystem properties for each individual forcing mechanism in the text. We request a further explanation on where / how the reviewer would like this expanded.

**Specific comments:**

**For Introduction**

**it should add more citations to support some sentences in the 2nd paragraph.**

We have included additional citations to support the role and characteristics of paleo records, experiments, and long term monitoring ecological studies to understand impacts of climate change in marine communities.

**Page 6, line 12, 'select' should be 'selected'**

“Select” is used here to indicate a specific group of ecosystems not as a verb.

**For Methods**

**Page 8, lines 5 t 6, missing a right bracket.**

Right parenthesis added.

**Page 9, line 4, 'living from' or 'living on'?**

Yes, this was awkward wording; changed to “subsisting on.”

**Page 15, line 9, grammatical error in 'Search Rate a behavior ..." missing a verb.**

Added “is.”

**For Results**

**Pages 17 - 18 (first paragraph), inappropriate interpretation: Looking at Fig. 2, I do not think they are generally consistent, and highlighting the differences is more important. For instance, though coralline algae did display consistent decrease, other groups (snails, lobsters) did not or at least showed high levels of sensitivity.**

The reviewer is correct in that ‘generally consistent’ does not accurately characterize the patterns observed. This has been changed to ‘directionally consistent’ with a reference to varying extents of change to highlight this difference. While this particular paragraph was designated to provide a quick overview of the results, it was not intended to get into the specific differences between forcing mechanisms, and a more detailed analysis of these differences can be found later on in the results section under ‘Comparing Forcing Types’. However, we did highlight potential differences between simulations in sentence 5 of the first paragraph and added in example species to further address the reviewer’s comment

**Page 20, 'Under Vulnerability forcing (Figure 2C), ...': I personally found the Vulnerability forcing result are not so similar to Production forcing one, and thus should be explained in much more detail.**

Changed ‘similar’ to ‘similar in direction’. The rest of the paragraph was edited to further highlight those specifics differences in more detail

**Page 20, ' except for brown algae, where the low-sensitivity scalar includes negative values despite the base result of a 20% increase in abundance. ': This is wrong. Other groups were also sensitive to the scalars. Such as pink abalone, black urchin, lobster.**

Made a number of revisions in the paragraph to more accurately reflect the directional changes in abundances and specifically highlighted the groups the reviewer pointed out.

**Page 21, lines 19 - 21 & Page 23, line 20 'not as significant as...': This might be wrong for specific groups like cabezon and pink abalone as shown by the figure. The explanation does not tell anything as it is just another way of narrating the result. I worry if the less prominent effect from other mortality forcing was driven by the potential poor choice of parameters to simulate this forcing in the model. At least, based on the methods by Busch and McElhany (2016), the mortality was linearly correlated with ph drop, which as the authors mentioned is not commonly found in nature. In addition, whether this empirical model could be used here in this study was not fully explained by authors. Meanwhile, as the authors also mentioned, shifts in temperature and dissolved oxygen could also interact with the pH but were not considered in this study. So the authors should at least acknowledge these potential contributing factors and interpret their results with serious caution and do not overclaim that direct mortality caused by OA is not significant as compared with other forcing types.**

The explanation on Page 23 was changed to ‘This was likely due to the fact that the additional mortality for each taxon resulting from OA was relatively small compared to the effects of predation and production on biomass through the other forcing types over this pH range.’

The reviewer is correct in his recommendation of caution when interpreting results based upon the mortality parameters presented by Busch and McElhany (2016). The parameters and relationships derived in Busch and McElhany (2016) were specifically developed for the use in ecosystem modeling studies, albeit via ATLANTIS, and therefore were the most up-to-date and applicable to our research. With respect to incorporating those mortality parameters into our model, we followed the methods of Marshall et al. (2017), which used the scalars from Busch and McElhany (2016) in an empirical equation solving for mortality of invertebrates that resulted in rates consistent with the literature. We actually used a higher scaling factor than Marshall et al. (2017) to achieve the same proportional increase in mortality resulting from pH. These methods are described on page 16.

The alternative was to recreate our own literature review across all species and groups, but we still would have been faced with the question of how to handle nonlinear responses due to a lack of overall information in the literature quantifying mortality rates at varying pH levels. As such, we felt that Busch and McElhany (2016) handled that gap in information responsibly. There is a similar gap with respect to the interacting effects of temperature and dissolved oxygen and we address these unknowns and their potential consequences in the last paragraph on page 29. However, it is unknown whether these interactions would also increase the impacts of OA through the Production and Vulnerability mechanisms along with Mortality. As such, we cannot claim that mortality would have a comparatively more significant effect. The same principle applies to the consequences of using linear scaling relationships.

Furthermore, from our experience using a variety of ecosystem models, small changes in the production of low trophic level groups have disproportionately large impacts on the biomass of groups in the ecosystem when compared to increases in top-down mortality of upper trophic species, such as through increased fishing pressure. Therefore, the results of this study are consistent with other modeling research we’ve seen in the past.

**Page 21, line 24: Confusing on 'linearly consistent'. Meanwhile, though it might be consistent with some groups, but definitely not for others (e.g. basses) that had different responses to different forcing factors.**

‘Linearly consistent’ was changed to ‘showing additive changes across the individual impacts of each forcing type’, which is a more accurate description and takes into account the directional differences displayed in some groups across simulations.

**Page 23, line 15: Again, I disagree that they are generally consistent. It is more important and interesting to highlight the difference, which was shown by the figure.**

Revised the sentence to ‘generally consistent in shape and magnitude across forcing types’. We believe this to be the case because the overall directional trends and shapes of the relationships do not drastically differ between forcing types. When taking into account the annotated scale on the y-axis, we also believe that the magnitude of each trend does not drastically differ.

But with that being said, the rest of the paragraph delves into the differences between forcing types in general, and the 4 paragraphs before this breakdown those differences at a more detailed level with respect to each ecosystem property.

**Page 24, line 1 'nonlinear amplifications': This would be better supported with a specific analysis rather than a superficial statement.**

Per the reviewer’s suggestions, we conducted a series of nonlinearity tests (Kolmogorov-Smirnov) where we 1) calculated the differences between the baseline model and time series for each forcing mechanism, 2) added those differences together, 3) calculated the difference between those added time series and the difference between the Combined simulation time series and the baseline model, 4) then conducted a Kolmogorov-Smirnov test on the linearity of those final time series.

|  | K-S Statistic | P-value |
| --- | --- | --- |
| Biomass | 0.4054 | 1.24E-86 |
| Commercial Biomass | 0.4417 | 9.81E-103 |
| Biodiversity | 0.4989 | 5.85E-131 |
| Trophic Level | 0.4993 | 3.45E-131 |

Results show that there are distinct nonlinear shifts between how the Combined mechanisms shift ecosystem properties versus the additive shifts from each individual forcing mechanism.

The above table was added into the manuscript and the following was added into the text

Methods: ‘Kolmogorov-Smirnov tests were also used to test for nonlinearity in the differences between the additive effects of each individual forcing mechanism and the combined simulation. The time series of differences were derived by first calculating the differences between the baseline model values and time series for each forcing mechanism, adding them together, and then calculating the difference between those added time series and the difference between the Combined simulation time series and the baseline model.’

An additional table was added into the results at the end of the ‘Ecosystem Responses’ section presenting these results.

**For Table 2, the authors misplaced the unit in a few places. For instance, Gross efficiency (catch/net p.p.) should be unitless, while Calculated total net primary production should have a unit.**

Units corrected for Gross Efficiency and Calculated Total Net Primary Production

**Reviewer #2: Review for: “Forecasting ocean acidification impacts on kelp forest ecosystems” by Schlenger et al. Overall, the paper was well written and understandable.**

**Using ecological models to integrate results from different methods. Thus leading to an assessment of Ocean Acidification on kelp community structure. Better understanding of food web dynamics responding to external forcing parameters. Present a quantitative modeling approach to asses the cumulative effects of multiple OA impacts on ecosystem dynamics. Results reveal that impacts from OA is strongly influenced by specific physiological interactions of OC with specific functional groups and species.**

**Overall comments**

**Overall, the manuscript is well written and the research is interesting and validates previous similar studies.**

**I recommend to the authors to read and include the Minireview by Harley et al. 2012 “Effects of climate change on global seaweed communities” – it has some very relevant insight.**

We thank the reviewer for highlighting this paper. We have incorporated some of its ideas into parts of the Introduction and Discussion.

**To bottom of page 27- the authors could add more detailed data from Kubler et al. 2015 PLoS ONE “Predicting effects of ocean acidification and warming on algae lacking carbon concentrating mechanisms.”**

Although we agree that Kubler and Dudgeon (2015) is an interesting and relevant paper, we chose to discuss their results in the paragraph about interactions with temperature rather than the paragraph suggested by the reviewer since it seemed more appropriate there.

**Specific comments**

**A map of Isla Navidad would be useful to add to the intro – under “ecopath Model”**

We have included a map of the island indicating the location in relation to North America.

**Figure 1 – the text is very blurry – please make the font bigger and more legible. Figure 2 is also blurry – please make more legible.**

**Figure 1 (now Figure 2) has been recreated to be more clear**

**Table 1 . “Sea palm” usually refers to Postelsia palameformis. Curious on why do you separate sea palm from brown algae and Sargassum categoryTechnically, Sargassum, Macrocystis, Ecklonia are all brown algae too. Throughout the text and in your figures, you predominantly refer to brown algae – so it is not clear what groups you include. Perhaps there is a better way to make the distinction?**

To avoid further confusion with this group, we have changed the name from “Sea palm” to  “Sub canopy kelp”. We initially used this term as *Ecklonia arborea* is known as “The Southern Sea Palm”. Additionally in table 1, we have listed the genus and species for the most representative groups for all functional groups in the model.

**Pg 18 – missing % after 15.**

Added “%.”

**Figure 2 – why only “other mortality” where is overall mortality (natural + predation +fishing +other)? Is it included in Vulnerability?**

Total Mortality (described on page 15) is a combination of natural mortality, predation mortality, fishing mortality, and other mortality, which are all calculated in the EwE model. Other mortality is a subcomponent of this calculation and is handled separately. In order to further emphasize this point, we added in the following test to page 15.

‘Total Mortality is derived in EwE through a variety of natural growth parameters, predation rates, and fishing rates, while the contribution of Other Mortality can be manually added to this calculated at any timestep.’
