# Supplementary material for: Forecasting ocean acidification impacts on kelp forest ecosystems
Source: PLoS One. 2021 Apr 22;16(4):e0236218. doi: 10.1371/journal.pone.0236218 (PMC8061940; doi:10.1371/journal.pone.0236218)
Supplement: Attachment — Submitted filename: Schlenger et al. Response to Reviewer Comments.docx [file pone.0236218.s003.docx]

**Schlenger et al. Response to editor comments**

Line 76 – please remove contractions here and anywhere else they occur.

Corrected this and a handful of other instances of contractions throughout the paper

Line 114 – you will need to add in “non-calcareous”, as the impacts of OA on coralline algae, Halimeda, etc are certainly not positive.

Added

Line 178 – Reference 46 is a kelp forest ecosystem

Included

Line 233 – This needs to reference the numbered references in the references section please. Please check elsewhere also for these instances.

Numbers were included alongside all references

Line 547 – Cornwall and Eddy.

Fixed

Line 570 – This sentence does not make sense entirely, because in Cornwall and Eddy there was only fishing presence/absence (i.e. MPA or not) and OA presence/absence. Do the authors mean OA had a larger effect in the absence of marine protection?

What we meant to say here is that the effects of OA were larger on groups when compared to the effects of fishing pressure in Olsen et al whereas the effects of fishing were larger than OA in Cornwall and Eddy. Therefore we removed ‘and marine protected areas’ from the sentence to just focus on OA and fishing pressure. The sentence now reads as

“In general, OA had a larger impact than fishery pressure (contrary to Cornwall and Eddy 2015).”

Line 579 – pH 6.7 likely does not represent a scenario we will ever observe in most marine habitats due to OA.

That is true and it is why our study did not include such an extreme pH. However, we’re reporting the results of Porzio et al. (2011) to provide evidence of ecological changes along an extended pH gradient.

Line 628 – Remove the words” results showed that”.

Removed

Line 654 – I consider that any chemical effect of temperature will be very minimal compared to the impact of temperature on organism physiology.

The sentence has been updated to read as

“Aside from the direct impact of temperature on organismal physiology, increasing temperature will also exacerbate the effects of low pH by stratifying the water column and further decreasing calcium carbonate availability as well as inhibiting reoxygenation of the water column and further decreasing metabolic capacity [77]”

Figures: Figure 3 and 4 appear to be straight form excel, it would be ideal if the authors could improve these.

The figures have been reproduced using Sigma Plot. Please let us know if you would like to see any further changes.

References: Some of these require formatting, for example, if title names are listed in all capital letters, this needs to be addressed. Please go through each reference to check formatting.

Fixed
